# Optimization of transcription factor binding map accuracy utilizing knockout-mouse models

**DOI:** 10.1093/nar/gku1078

**Published:** 2014-11-05

**Authors:** Wolfgang Krebs, Susanne V. Schmidt, Alon Goren, Dominic De Nardo, Larisa Labzin, Anton Bovier, Thomas Ulas, Heidi Theis, Michael Kraut, Eicke Latz, Marc Beyer, Joachim L. Schultze

**Affiliations:** 1 Genomics and Immunoregulation, LIMES-Institute, University of Bonn, 53115 Bonn, Germany; 2 Broad Institute of MIT and Harvard, Cambridge, MA 02142, USA; 3 Institute of Innate Immunity, University Hospitals, University of Bonn, 53127 Bonn, Germany; 4 Institute for Applied Mathematics, University of Bonn, 53115 Bonn, Germany; 5 Division of Infectious Diseases and Immunology, UMass Medical School, Worcester, MA 01605, USA; 6 German Center of Neurodegenerative Diseases (DZNE), 53175 Bonn, Germany

## Abstract

Genome-wide assessment of protein–DNA interaction by chromatin immunoprecipitation followed by massive parallel sequencing (ChIP-seq) is a key technology for studying transcription factor (TF) localization and regulation of gene expression. Signal-to-noise-ratio and signal specificity in ChIP-seq studies depend on many variables, including antibody affinity and specificity. Thus far, efforts to improve antibody reagents for ChIP-seq experiments have focused mainly on generating higher quality antibodies. Here we introduce KOIN (knockout implemented normalization) as a novel strategy to increase signal specificity and reduce noise by using TF knockout mice as a critical control for ChIP-seq data experiments. Additionally, KOIN can identify ‘hyper ChIPable regions’ as another source of false-positive signals. As the use of the KOIN algorithm reduces false-positive results and thereby prevents misinterpretation of ChIP-seq data, it should be considered as the gold standard for future ChIP-seq analyses, particularly when developing ChIP-assays with novel antibody reagents.

## INTRODUCTION

Genome-wide localization of transcription factors (TF), chromatin regulators, histone modifications and histone variants is mainly assessed by ChIP-seq, establishing it as a central technology for understanding transcriptional regulation in living cells (1,2). The precision of ChIP-seq experiments and their subsequent correct biological interpretation relies on many different parameters, including chromatin fragmentation, antibody affinity and specificity, DNA library preparation, genomic coverage of sequencing reads, sequencing depth and computational algorithms for peak calling (3–5). Since antibody quality is critically important for successful ChIP-seq experiments, immunoblotting (4) or ChIP-string methodologies (6,7) are used to define the affinity and specificity of antibodies used in ChIP-seq experiments. In addition to specifically enriched sites with biological relevance ChIP-seq data can also contain none relevant but specific signals due to the cross-reactivity of antibodies used for ChIP-seq experiments against proteins other than the epitope used for immunization. However, ChIP-seq data can also include random signals widely distributed over the whole genome, which are normally dismissed as background noise. These signals not only vary in binding motifs but also in signal intensities and are believed to originate from unspecific binding of DNA to beads or to the constant FC region of antibodies. Therefore, it remains difficult to distinguish such false-positive signals from true TF-associated peaks, especially in cases of low enrichments for binding motifs at called peak positions (8). Interestingly, false-positive peaks were even called in ChIP-seq experiments performed against a protein without a DNA-binding domain (9). In addition, technical aspects like shearing efficiency or crosslinking procedures can generate false-positive signals (4). Another ChIP-seq-specific variance are so-called ‘hyper-ChIPable regions’, recently described in yeast (9). High levels of transcription have been linked to these euchromatic sites with large numbers of ChIP-seq-binding signals enriched at these sites. So far, nucleosome depletion at transcriptional active sites is considered to expose the DNA in a greater extent to beads and antibodies during immunoprecipitation. Apparently this susceptibility leads to unspecific precipitation of DNA during ChIP-seq experiments. Due to these limitations, we postulated that employing TF knockout (KO) cells in ChIP-seq experiments should significantly increase signal-to-noise ratios by correcting for background signals, and should increase signal specificity by allowing for correction of peaks originating from nonspecific antibody-protein binding. Based on this hypothesis, we utilized ChIP-seq data from TF-KO control samples to develop a novel approach, called the Knockout Implemented Normalization (KOIN) method to reduce false-positive signals, identify ‘hyper-ChIPable regions’ and significantly improve biological downstream interpretation. We utilized six freely available ChIP-seq TF data sets (10–13) to demonstrate that KOIN increases the precision of ChIP-seq data interpretation for each data set.

## MATERIAL AND METHODS

### ChIP-Seq data sets

The data sets for ATF3 (GSE55317) were generated from bone marrow-derived macrophages (BMDMs) as previously described (10). In brief, BMDMs from 6- to 8-week old wild-type (WT) C57BL/6 and ATF3-deficient mice were obtained by culturing bone marrow cells for 6 days in DMEM supplemented with 10% (vol/vol) FCS, 10 μg/ml Ciprobay-500 and 40 ng/ml M-CSF (R&D Systems). BMDMs were pretreated with medium alone (unstim), 2 mg/ml HDL for 6 h or 2 mg/ml HDL for 6 h followed by stimulation with 100 nM CpG for 4 h. ChIP-Seq experiments for GATA3 (11) (GSM523224/GSM742022), SRF (12) (http://homer.salk.edu/homer/data/index.html; ‘SRF’ data set; http://homer.salk.edu/homer/data/ucsc/asullivan-10-12-01/ThioMac-SRF.fastq.gz; ‘SRF in SRF -/- mice’ datset; http://homer.salk.edu/homer/data/ucsc/asullivan-10-12-01/ThioMac.SrfKO-SRF.rep2.fastq.gz) and PU.1 (13) (GSM538003/GSM537999/GSM538000) including library preparation and base-calling are described in the corresponding publication. The alignment to the NCBI Build 37 genome assembly (mm9) was carried out for all data sets with Bowtie (14). Bowtie alignment was performed with the following settings for concatenated replicate data sets: -t -q -e 70 -l 28 -n 2 -best -maxbts 125 -S. All reads mappable to the reference genome were used for downstream peak calling including multi-mappable read positions, determined by Bowtie. Scores for multiple alignments for every read were reported and only the position with the best score was further used in the analysis.

### KOIN pipeline

KOIN-corrected peak files were generated with the KOIN pipeline. ChIP-seq data sets for WT and KO experiments were first aligned to a reference genome with the Bowtie program. Aligned sam/bam files were afterward used for peak calling with MACS. During KOIN, the KO data set was used as a control to call false-positive curated peak positions for the WT data set. Next peak positions with normalized tag counts in WT to KO data sets with fold changes smaller than 2 were filtered out. These data are then used for downstream data analysis like annotation and PWM motif enrichments using HOMER (13). The KOIN method is provided as a command line-based batch process to be operated in a linux environment (see also Supplementary Methods) which is available online (https://github.com/LIMES-NGS/KOIN-pipeline).

### Peak identification for standard and KO implemented normalization (KOIN) method

MACS (Model-based Analysis of ChIP-Seq) v1.4.0/v2.0.1 (15) peak calling was performed with the following options: -g 1.87 × 10^9^ -s 51 -bw 150 -w -single-profile -p 1 × 10^-4^ -on-auto. In brief, WT peaks used in the standard method were called using MACS v1.4.0 with aligned ‘.bam’ files as input. Effective genome size was set to 1.87 × 10^09^. Intervals on the reference genome with bimodal signal enrichments were defined as peak regions, with strand tags enriched upstream and anti-sense strand tags enriched downstream of TF binding. MACS shifted sense and anti-sense tags to the peak midpoint and detected tag enrichments at peak positions. To filter out peak positions without significant tag enrichments, MACS used a Poisson distribution model with the parameter (λ_local WT_).

\begin{equation*} \lambda _{{\rm local}\,{\rm WT}} = \max (\lambda _{{\rm BG}\,{\rm WT}} ,\lambda _{5{\rm k}\,{\rm WT}} ,\lambda _{10{\rm k}\,{\rm WT}} ) \end{equation*}

MACS defined λ_local WT_ as the maximum background signals. This is calculated by MACS either from the whole WT data set (λ_BG WT_) or from 5- or 10-kb regions centered to the peak midpoint (λ_5k_, λ_10k_) depending on the maximum signal. The sum of called WT peaks included all peaks which fit to the Poisson distribution model with *P*-values smaller than 0.04.

When utilizing data from KO mice (KOIN method), aligned ‘.bam’ files from WT experiments were used as ‘treatment’ file and aligned ‘.bam’ files from KO experiments as ‘controls’ to calculate background signals utilizing the MACS algorithm. Peak calling steps were performed according to standard method described before. After the first step, the sum of WT peaks included all peaks, which showed *P*-values smaller than 0.04 fitting to a Poisson distribution model based on the parameter λ_local KO_.

\begin{equation*} \lambda _{{\rm local}\,{\rm KO}} = \max (\lambda _{{\rm BG}\,{\rm KO}} ,\lambda _{{\rm 1k}\,{\rm KO}} ,\lambda _{{\rm 5k}\,{\rm KO}} ,\lambda _{{\rm 10k}\,{\rm KO}} ) \end{equation*}

λ_local KO_ is defined as the estimated maximum background signal. This is calculated by MACS either from the whole KO data set (λ_BG KO_) or from 1-, 5- or 10-kbp regions centered to the peak summit (λ_1kKO_, λ_5kKO_, λ_10kKO_) depending on the maximum signal. In the next step, ‘treatment’ and ‘control’ files were swapped and a second peak calling was performed for KO peaks (defined as ‘negative’ peaks) found in the KO data set. All KO peaks are called fitting with *P-*values smaller than 0.04 to a Poisson distribution model with the parameter λ_local WT_. λ_local WT_ was defined as estimated maximum background signals in the WT data set. This is calculated by MACS either from the whole data set (λ_BG WT_) or at different regions with 1-, 5- or 10-kb length around the peak summit (λ_1kWT_, λ_5kWT_, λ_10kWT_).

\begin{equation*} \lambda _{{\rm local}\,{\rm WT}} = \max (\lambda _{{\rm BG}\,{\rm WT}} ,\lambda _{{\rm 1k}\,{\rm WT}} ,\lambda _{{\rm 5k}\,{\rm WT}} ,\lambda _{{\rm 10k}\,{\rm WT}} ) \end{equation*}

Nonspecific peaks present in the intersection between WT and KO peaks called in the first two steps of the KOIN method were excluded for further analysis. Finally, the sum of corrected peaks after the KOIN method only contained significant WT peaks as described in the following formula:

\begin{eqnarray*} &&{\rm Corrected}\,{\rm peaks}({\rm KOIN}\,{\rm method})\nonumber \\ &=& {\rm WT}\,{\rm peaks}\backslash ({\rm KO}\,{\rm peaks} \cap {\rm WT}\,{\rm peaks}) \end{eqnarray*}

To increase specificity, peaks with less than 2-fold higher signals observed in normalized WT tag counts in comparison to KO tag counts at called peak positions were excluded. The module ‘annotatePeaks.pl’ included in the HOMER program can count and size-normalize ChIP-seq tag counts located at each peak position in WT and KO data sets specified with the -d option (command: annotatePeaks peak-file.bed mm9 -size given -d peak-file-tag-directory/). Every statistical software suit, for example, SPSS or Excel can then calculate the fold difference between normalized tag counts for WT compared to KO data sets.*P*-values were adapted from the standard value 0.05 to 0.04 to reduce the number of enriched regions to a level allowing statistical analysis with the GREAT tool.

Verification of peak identification with MACS was performed with a second peak calling algorithm called SICER (Spatial clustering for Identification of ChIP-Enriched Regions) v1.1 (16). For the standard approach in SICER, the following options were used: mouse reference genome mm9, redundancy threshold 1, window size 100 bp, fragment size 150 bp, effective genome fraction 0.77, gap size 100 bp and *E*-value 100 (16). Files in ‘.bed’ format were loaded as input into SICER and clonal tags were excluded from the analysis. Next, SICER shifted tags aligned to the sense and anti-sense strand for half of the fragment size and identified significantly enriched islands with the specified windows size and a window score. All significant WT peaks called with the standard method required scores above the window score threshold and an *E*-value less than the threshold based on the random background model used.KOIN peak calling utilizing KO data sets as controls was performed with the options: mouse reference genome mm9, redundancy threshold 1, window size 100 bp, fragment size 150 bp, effective genome fraction 0.77, gap size 100 bp, *E*-value 100 and a false discovery rate (FDR) of 0.01. During KOIN, WT peaks were first called according to the standard method described. These candidate peaks were further filtered according to the *P-*value threshold under a Poisson distribution model defining WT data set read counts and KO data set read counts as parameters and using Bonferroni corrections for multiple testing. The numbers of ChIP read counts in WT and KO data sets for each peak were adapted to the total ChIP library size. Finally, corrected peaks with false discovery rates of < 0.01 based on the Poisson *P-*value were used for further downstream calculations.

To determine the relative overlap of peaks called by SICER and MACS, we applied the Hypergeometric Optimization of Motif EnRichment program (HOMER) v4.3 (http://homer.salk.edu/homer/index.html) (13). The mergePeaks module in HOMER was used for the comparison of two different peak files resulting in the identification of common and unique peak positions (command: mergePeaks MACS-peaks-file.bed SICER-peaks-file.bed -d given).

### Annotation and fragment enrichment analysis

Peaks were annotated to the mouse reference genome (mm9) according to Refseq TSS information using HOMER. Annotated peak positions were used to detect global peak distributions in standard or KOIN-corrected data sets. WT and KO ChIP-fragment signals were depicted as histograms 2 kb up- and downstream of each peak center in 10-bp sliding windows in histogram mode and were normalized before analysis to 10^7^ total tag numbers. Java-Treeview (v1.1.6.r4) was used to create heatmaps. For graphical display of the ATF3 enriched example regions, normalized ChIP-seq tag signals and peak locations were loaded into integrative genomics viewer IGV (17). This approach allowed the comparison of WT and KO ChIP-seq tag signals at specific locations independent of total tag numbers in each data set.

### Motif discovery

Peak files calculated by standard or KOIN method by MACS were used for TF motif discovery by HOMER. For comparison, peak sites identified as false positives were also examined for motif predictions. TF-binding sites in a region of 100 bp up- and downstream of peak centers were used to isolate sequences as input enabling a *de novo* motif analysis with HOMER. For GC normalization purposes, random background sequences with similar GC% contents were chosen. Auto-normalization was performed to remove imbalances in short oligos between target and background sequences. After search and optimization steps, motifs were identified. Top 10 HOMER motifs were chosen, according to their binomial *P*-values, from each data set and the *P-*values were depicted in heatmaps by Partek genomic suite (v6.6). *P*-values were converted with the following formula into positive numbers representing the statistical significance of motif enrichment: -log10 (motif *P*-value). Percentages of target sequences with corresponding binding motif determined by the standard method or the KOIN method were compared and depicted with horizontal aligned bar plots together with the corresponding positional weight matrices (PWM). For each motif, the percentage defines the number of ChIP-seq tags with occurrence of the particular motif among all tags. Multiple motif hits at a given tag site are possible.

Motif ratios were calculated for the top five enriched motifs called independently in WT and KO data sets with HOMER by counting the motif occurrence and dividing the motif numbers in the WT data set by the numbers in the KO data set. Motif counts were normalized against total peak counts for every experiment. Higher motif abundance in WT compared to KO data sets were defined as positive fold change values and vice versa for higher ratios in KO compared to WT data sets with negative values.

### GO-term enrichment analysis for proximal regions

For every peak, the nearest TSS was identified. Corresponding gene names of peaks at promoter sites within 1000 bp of TSS respectively peaks inside gene loci were loaded into Cytoscape v2.8.3 (18) with the BiNGO (19) plugin (v2.44) and gene set enrichment was performed using an FDR threshold of 0.001. Visualization of the Gene Ontology (GO) analysis as networks was performed with the plugin Enrichment Map for Cytoscape (v1.2). The cutoff for the Jaccard coefficient was set to 0.001 and an FDR *Q*-value of 0.01. KOIN-corrected and noncorrected results were overlaid to visualize enriched GO-terms after correction. Clusters of GO-terms were marked and named according to common functions filtered by the plugin word cloud. Only subnetworks with greater than four GO-terms are shown.

### Differential GO-term enrichments including distal loci

We used the Genomic Regions Enrichment of Annotations (GREAT) tool v2.0.2 (20) to study functions of sets of noncoding *cis*-regulatory genomic regions and TSS-associated regions. MACS peaks were associated with their putative target genes and connected to corresponding gene ontologies by GREAT. After statistical enrichment, calculations with binomial and hypergeometric test algorithms GREAT displays significantly enriched GO-terms. GO-terms with and without false-positive corrections were compared against each other according to their binomial *P*-values.

### Identification of ‘hyper-ChIPable regions’

The top 25 TF enriched peak positions were ranked according to their normalized tag counts independently for all six data sets. Only loci with occurrence in at least two data sets were used for detailed inspection with UCSC genome browser (http://genome.ucsc.edu). To define these loci as ‘hyper-ChIPable’, the following criteria were used: potential regions display additional enrichment for unrelated DNA-binding proteins currently available in the ENCODE database (described below) and are also characterized as DNAse I hypersensitive sites; regions show RNA polymerase II binding and histone modifications including H3K4me1, H3K4me3 and H3K27me3. Potential ‘hyper-ChIPable regions’ were analysed visually in all data sets (https://genome.ucsc.edu/ENCODE). In case the investigated regions showed high signals beyond the viewable default scaling, the region was defined as ‘hyper-ChIPable’. The following data sets were used: TF-binding sites (Caltech TFBS; LICR TFBS; PSU TFBS; Stan/Yale TFBS), histone modifications (Caltech Histone; LICR Histone, PSU Histone; Stan/Yale Histone), DNAse I hypersensitive cleavage sites (PSU DNaseI HS; UW DNaseI DGF; UW DNaseI HS).

## RESULTS

### KOIN reduces false-positive signals in ChIP-seq data

**Figure 1.**
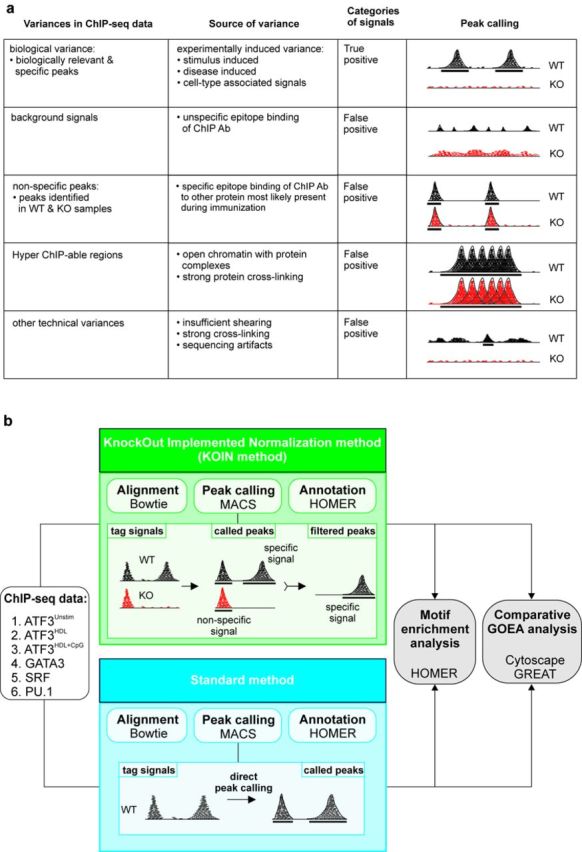
Sources of variance in ChIP-seq experiments and schematic overview of the KOIN method. **(a)** Overview of factors influencing ChIP-seq data. **(b)** Schema of two approaches for analysis of ChIP-seq data using either the KOIN method (KO Implemented Normalization method, in green) including knockout (KO) samples or the standard method (in blue). Next generation sequencing data were aligned to the reference genome using Bowtie; peaks were called with MACS (preferred peak caller) and annotated to the transcript database using HOMER. Results for both methods were compared during downstream data processing including motif enrichment analysis using HOMER or comparative gene ontology enrichment analysis (GOEA).

A major prerequisite for correct interpretation of ChIP-seq data is the reduction of false-positive signals to a minimum (4). Moreover, while true positive signals are derived from the biology of the sample (biological variance, Figure 1a), additional false-positive signals derived from different types of variance, such as background signals, nonspecific peaks, ‘hyper-ChIPable regions’ and other technical variances can exist (Figure 1a). We postulated that the particular background signals generated by nonspecific peaks and ‘hyper-ChIPable regions’ result in false-positive signals, which should be eliminated when subtracting signals in ChIP-seq data of transcription factor (TF) knockout (KO) samples from those obtained using wild-type (WT) samples. We termed this approach Knockout Implemented Normalization (KOIN). Major steps necessary to perform the KOIN calculations are described in the KOIN pipeline (Supplementary Figure S1). We evaluated the advantages of KOIN in comparison to the standard method utilizing six different ChIP-seq data sets (10–13) assessing global binding patterns of the TFs PU.1, SRF, GATA3 and ATF3 (Figure 1b). In addition, we also compared downstream data analysis like motif enrichment or comparative gene ontology enrichment analysis (GOEA)_ENREF_6. As the primary peak caller, we applied MACS v1.4.0, while MACS v2.0.1 showed similar results (data not shown) (15). In essence, KOIN includes as a first step peak calling in WT followed by removal of peak regions that are called in both WT and KO samples, and finally, regions with background signals identified in both WT and KOIN are eliminated (for more detail see material and methods).

**Figure 2.**
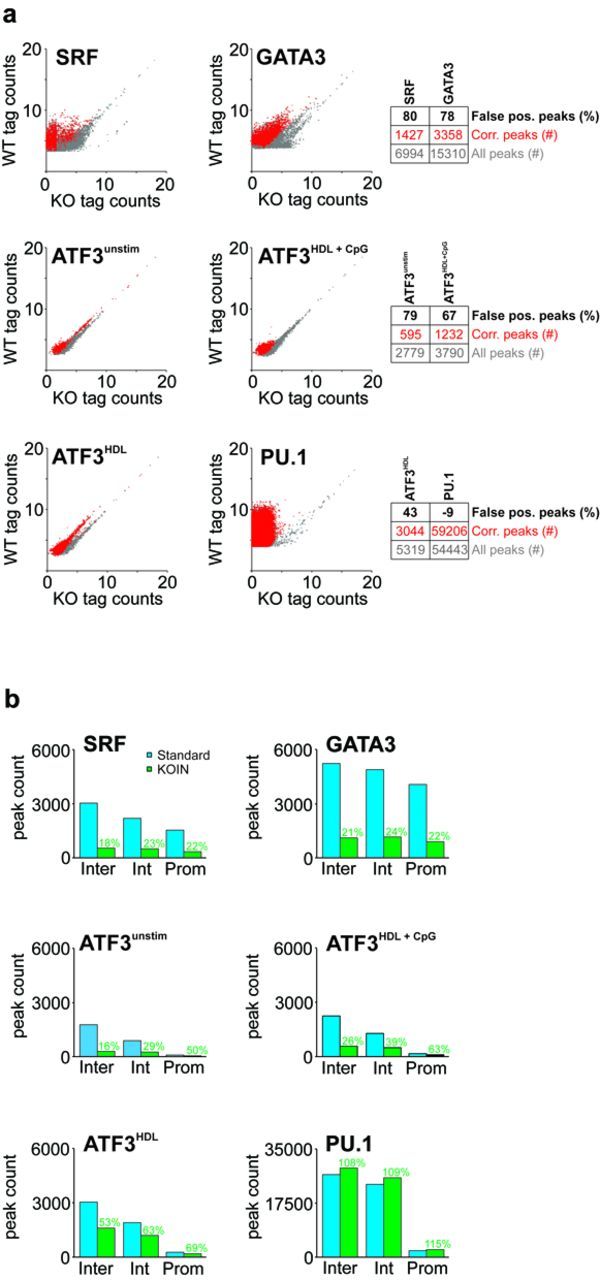
Global TF-binding distributions in independent ChIP-seq experiments reveal distinct false-positive signals. **(a)** Normalized ChIP-seq tag counts (log_2_ scale) for PU.1, SRF, GATA3 and ATF3 (under indicated stimulatory conditions) of uncorrected data derived from WT samples are plotted against tag counts derived from TF-KO samples (gray dots). Remaining peaks after KOIN-correction are overlaid (red dots). To avoid overlaps of a high fraction of data points Jitter was added. Numbers of total peaks, KOIN-corrected peaks and the false-positive peak rate (in percent) are presented as a table next to the dot plots. **(b)** Peak counts at promoter (Prom), intronic (Int) and intergenic (Inter) genomic regions are compared before (blue) and after (green) KOIN correction. Percentages of remaining peak numbers after correction (true positives) are included (green).

To investigate the degree of false-positive peaks, normalized tag counts for peaks were determined and tag counts in WT samples (gray color) were plotted against KOIN-corrected tag counts (red color) at the corresponding genomic location for the four TFs investigated (Figure 2a). Our method revealed a significant number of false-positive peaks in the SRF and GATA3 data sets. Interestingly, when addressing genome-wide DNA-binding maps of ATF3 in macrophages cultured under three different stimulatory conditions (unstimulated, stimulated with HDL, or HDL + CpG), the number of false-positive peaks for ATF3 was dependent on the cell culture condition. The correction of false-positive peaks (correction rate) led to a decrease in peak counts of 80% in the SRF, 78% in the GATA3 and from 43 to 69% in the ATF3 data sets (Figure 2a, Supplementary Table S1) while there was a slight increase of peaks called for PU.1 after correction (see below). For SRF and GATA3, we observed similarly high correction rates by KOIN when intergenic, intronic or promoter sequences were analysed separately (Figure 2b). Interestingly, when addressing genome-wide DNA-binding maps of ATF3 in macrophages cultured under different stimulatory conditions, differences of false-positive peak numbers for ATF3 were identified. In unstimulated cells and cells stimulated with HDL and CpG, a similar rate of false-positive peaks was observed in intergenic and intronic peaks, while peaks in promoter regions showed higher specificity as indicated by a higher rate of true positives (unstimulated cells: 50%, cells stimulated with HDL + CpG: 63%). In contrast, there was less difference between intergenic (53%), intronic (63%) and promoter peaks (69%) in cells stimulated with HDL alone.

To ensure that the number of false-positive peaks is not related to mapping quality as defined by the ratio of unique to multi-mappable reads (21), we determined the fraction of unique mapping reads for all six data sets and plotted them against the number of reads per position (Supplementary Figure S2a). First, concerning multi-mappable reads there was no difference between any of the corresponding WT and KO data sets. Second, no obvious pattern was revealed for false-positive rate and mapping quality, particularly with PU.1 and GATA3 showing similarly high fractions of uniquely mapping reads while showing rather different false-positive rates. Therefore, we kept multi-mappable reads for further analysis following previous approaches (21).

We next combined the peak caller SICER (16) with KOIN to see whether we could further improve our results obtained when using MACS as the primary peak caller. SICER revealed up to 90% of the peaks identified by MACS in uncorrected data with SRF showing the lowest overlap (Supplementary Figure S2b). Although the overlap between MACS and SICER was improved after KOIN-correction for the SRF data set, it still scored lowest in comparison to all other data sets. Overall, MACS seemed to outperform SICER, at least for the data sets analysed here.

### Global visualization further illustrates the loss of false-positive peaks

**Figure 3.**
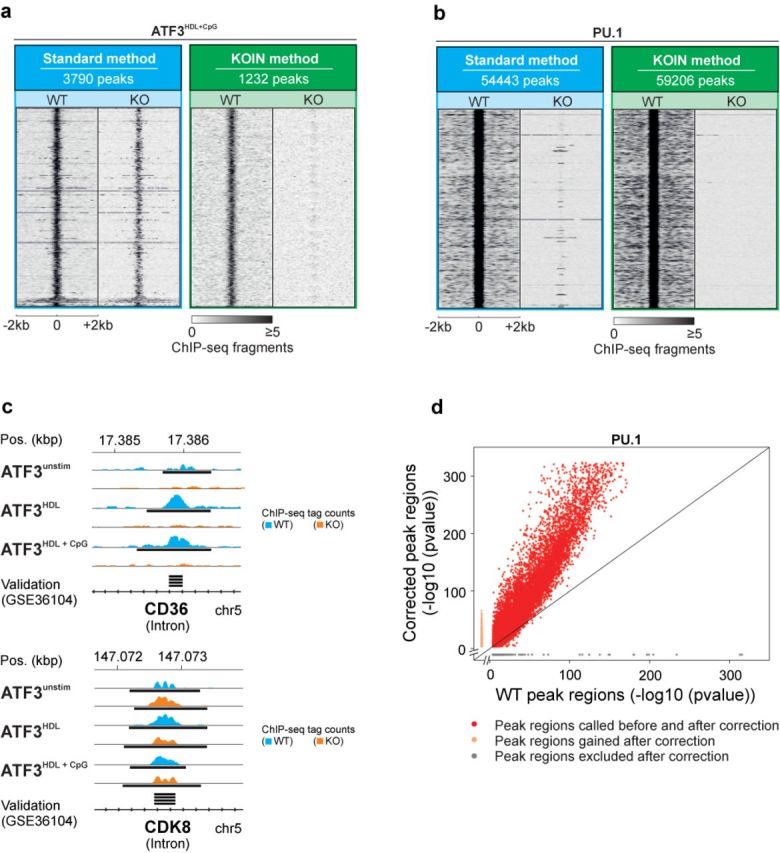
Global visualization of data set-specific effects on peak calling before and after KOIN correction. Heatmaps of normalized ChIP-seq tag counts centered at peak midpoints depicted for ± 2-kb windows for **(a)** ATF3 under the stimulatory influence of HDL and CpG as well as **(b)** PU.1 visualized for WT and KO samples separately before and after KOIN correction. A 10-bp sliding window was used to calculate tag densities and resulting numbers were normalized to 10^7^ total tag counts. **(c)** Representative ChIP-seq reads in the introns of true and false-positive ATF3-binding sites. Black bars indicate significant peaks identified by MACS with *P*-values ≤ 10^−4^. An independently generated ATF3 data set (6) was used to visualize peaks called significant (black bars) in the previous study performed without the use of KO samples. **(d)** Peak *P*-values before and after KOIN correction. MACS peak *P*-values for WT PU.1 enriched positions (x-axis, -log10 (*P*-value)) are plotted against the *P*-values for corresponding KOIN-corrected peaks (y-axis, -log10 (*P*-value)); red, significantly called peaks belonging to the WT- and KOIN-corrected data sets; gray, false-positive peaks lost after KOIN correction; orange, peaks exclusively called after applying KOIN correction.

While the data presented in Figure 2 depict the overall numbers of corrected peaks and their relation to tag counts, we next wanted to visualize the correction in relation to the respective peak center. Using the ATF3 data set (HDL + CpG) as an example, peak-centered tag count heatmaps of data before (standard method) and after KOIN-correction clearly illustrate that false-positive peaks are completely eliminated (Figure 3a). Moreover, signals appearing more distant from the major peak signals in both WT and KO samples are also removed from the data. Similarly, even in a data set with high specificity like the PU.1 data set, the KOIN method depleted all false-positive signals (Figure 3b). We further validated the KOIN algorithm by comparing our results with a previously published data set using the same anti-ATF3 antibody (GSE36104) (6). Using the CD36 and the CDK8 loci as examples (Figure 3c), ATF3 peaks were called for both loci in both experiments, yet only the peak in the CD36 locus seems to be ATF3-specific since both ATF3 KO and WT samples showed peaks in the CDK8 locus. In summary, visualization of KOIN-corrected positions demonstrated the successful exclusion of false-positive signals even beyond the major peak positions.

### Improved signal-to-noise-ratios can increase the number of peaks after KOIN

Surprisingly, we identified increased peak counts (+9%) in the PU.1 data set after applying the KOIN algorithm (Figures 2a and b and 3b). In sum, 6037 new peaks were called (Figure 3d, orange dots). Since KOIN also reduces background signals, the threshold for peak calling is further lowered. For data sets such as PU.1, characterized by a very low false positive rate, elimination of the small remaining background signals will lead to the identification of significantly enriched sites with low tag counts. With the elimination of only a low rate of false-positive peaks and the additional calling of peaks due to a changed threshold for peak calling, the number of positive peaks called by KOIN can actually exceed the number of peaks called by the standard method. At the same time, reduced background levels also increase the peak calling *P-*values as demonstrated by the shift of data points to the upper left corner of the diagram (Figure 3d, red dots). In conclusion, KOIN leads to a noise reduction and supports an optimized data analysis even for antibodies with high specificity, as exemplified for PU.1.

### KOIN eliminates ‘hyper-ChIPable regions’

**Figure 4.**
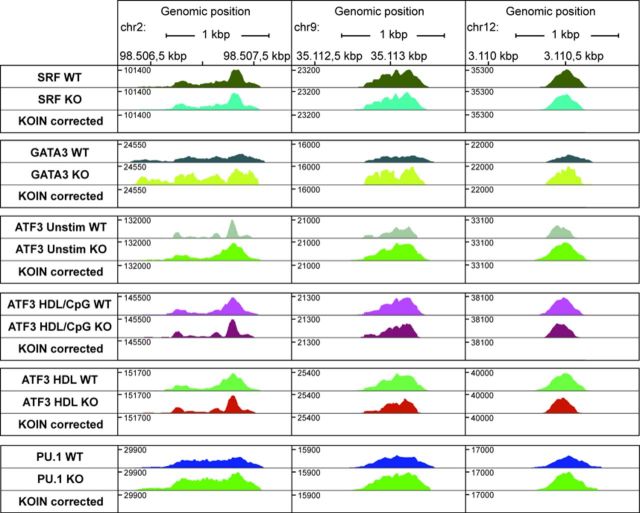
‘Hyper-ChIPable regions’ show extremely high ChIP-seq enrichments for all data sets. Three exemplary positions at ‘hyper-ChIPable regions’ were chosen for six ChIP-seq TF data sets (SRF, GATA3, PU.1 and ATF3 generated under different stimulatory conditions). Corresponding ChIP-seq profiles for WT and KO samples are depicted in different colors. In KOIN-corrected data sets, ‘hyper-ChIPable regions’ with corresponding tags are absent.

Another class of false-positive peaks known as ‘hyper-ChIPable regions’ have recently been discovered in yeast and are likely to occur in other species (9). These euchromatic loci are highly expressed in yeast and defined by a high enrichment of various unrelated DNA-binding proteins, strong RNA polymerase II binding, hypersensitivity to DNAse I cleavage and enrichment for certain histone modification marks (9). We focused on loci with the highest normalized ChIP-seq tag counts in at least two of our data sets and correlated them to the above-mentioned criteria using ENCODE resources (see material and methods). Indeed, we detected up to 18 ‘hyper-ChIPable regions’ in our data sets (Figure 4, Supplementary Table S2). However, more importantly, after KOIN-correction these regions were eliminated from the data set and the top 25 ranked peaks in the KOIN-corrected data did not fit the criteria of a ‘hyper-ChIPable region’ (Supplementary Table S3). These data indicate that our approach also accounts for and corrects false-positive peaks from ‘hyper-ChIPable regions’.

### KOIN enriches for peaks with binding motifs for the respective transcription factor

Next, we were interested if our approach had an impact on model building for TF-binding motifs as a complementary approach to evaluate peak calling specificity (4). We determined motif *P*-values, fractions of peaks with the respective motif and their changes between uncorrected WT, KO and KOIN-corrected data sets.

**Figure 5.**
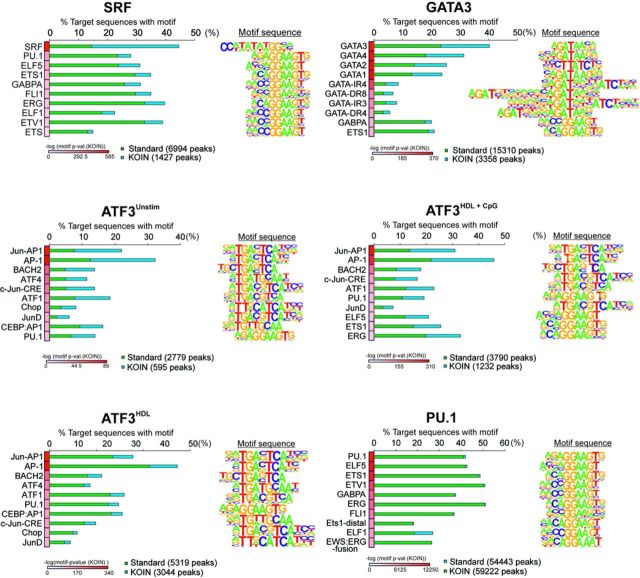
Impact of KOIN-correction on TF motif analysis. Top 10 significantly enriched TF-binding motifs in sequence elements found at SRF, PU.1, GATA3 and ATF3 binding sites were sorted according to their *P*-values after KOIN correction. The respective percentage of target sequences with corresponding motif are illustrated with (blue) or without (green) KOIN correction as horizontal aligned bar plots. Positional weight matrix (PWM) motif sequences are plotted at the right side of the corresponding motif. Corresponding *P-*values for each motif are plotted as heatmaps.

We identified highly enriched binding motifs for SRF, PU.1, GATA3 and ATF3 (Figure 5) for uncorrected and KOIN-corrected data using HOMER. Positive peaks for the top 10 TF-binding motifs were ranked according to motif *P*-values (after KOIN-correction, heatmap, from white to red) and the percentage of positive peaks for each of the motifs before (green) and after (blue) KOIN-correction were plotted. Due to the high specificity of the PU.1 data set, the percentages of peaks positive for TF-binding motifs remained unchanged (Figure 5). In contrast, we observed a profound relative increase in the corrected peaks for the SRF-binding motif (from 15 to 45% for SRF motif) with the lowest *P*-value in comparison to the nonspecific TF motifs and we observed the same trend for GATA3 and ATF3 (Figure 5). Moreover, when comparing all peaks called by MACS with those called by MACS and SICER neither the uncorrected nor the KOIN-corrected data sets showed a significant difference (Supplementary Figure S3a). Similarly, in five data sets the motif *P*-value (after KOIN-correction) for the main motif was lower in all peaks called by MACS versus those called by MACS and SICER (Supplementary Figure S3b), further suggesting that the peaks called by MACS followed by KOIN-correction truly reflect the global binding map of the respective TF. Motif enrichment analysis was also performed independently for KO data sets only (Supplementary Figure S4a). We observed an absence of the main motif in each data set, whereas other TF motifs were enriched with only low significance, indicating putative false positively called binding motifs. To further validate our motif-binding predictions, we compared the ratios of the top five enriched and independently called binding motifs for WT and KO data sets (Supplementary Figure S4b). In the SRF and PU.1 data sets, the main motifs were enriched. Due to the high similarity in the core binding sequence, not only GATA3 but also motifs for the whole GATA TF family were enriched in the GATA3 data set. Similar observations were made for the ATF3 data sets, in which the Jun-AP1 motif showed the highest enrichment ratios besides AP-1 or BACH2 for WT against KO data sets.

Taken together, these results suggest that false-positive peaks in the uncorrected data set can dilute the specific signal, and removal of these incorrect peaks provides an increase in percentage of peaks carrying the respective motif. Additionally, false-positive peaks can highly contribute to enrichment of binding motifs of unrelated TFs (Supplementary Figure S5).

### KOIN improves biological interpretation of ChIP-seq data

**Figure 6.**
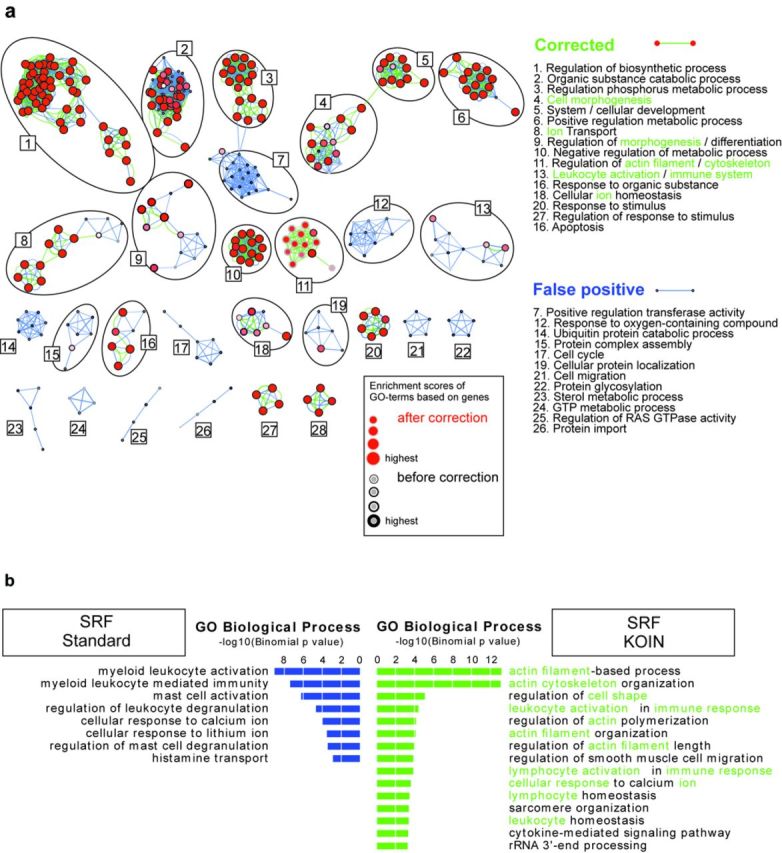
KOIN correction significantly changes GO-term enrichment analysis. **(a)** Network visualization of Gene Ontology Enrichment Analysis for genes with SRF protein binding signals located 1 kb up- and downstream from their TSS are visualized for 1510 genes before (black node borders: GO-terms, blue edges: GO-term relations) and 327 genes after the correction process (red nodes: GO-terms, green edges: GO-term relations). GO-terms correlating to genes remaining after correction procedure are depicted as red nodes with black borders. The binomial FDR corrected *P*-value cutoff was set to <0.001. Analysis was performed with Cytoscape and the two plugins BiNGO and Enrichment Map. **(b)** SRF ChIP-seq signals in *cis*-regulatory and noncoding genomic regions were analysed with the GREAT tool to determine biological relevance of ChIP-Seq-binding patterns. Enriched GO-terms passed threshold for FDR corrected *P*-values set to 0.05 with both binomial and hypergeometric tests. Depicted are the binomial *P*-values (-log10 (*P*-value)) of enriched GO-terms with (green) or without (blue) KOIN correction. Commonly found KOIN-corrected GO-terms in (a) and (b) are highlighted in green letters.

To illustrate the impact of reducing false-positive results via KOIN on biological interpretation of ChIP-seq data, we performed Gene Ontology Enrichment Analysis (GOEA) based on SRF TF peaks near the transcriptional start site (±1 kb away from TSS) and inside gene loci followed by network visualization of enriched GO-terms using BiNGO and EnrichmentMap (Figure 6a). This analysis revealed a profound reduction in the number of biological processes associated with the remaining gene loci in the KOIN-corrected data set. Out of the 13 major and 9 minor subnetworks of GO-terms, two major subnetworks (s7, s12) and 10 minor subnetworks (s14, s15, s17,s 19, s21-s26) were completely lost in the KOIN-corrected data set. While there is clear evidence for an involvement of SRF in those biological processes that remained in the KOIN-corrected data (12,22), there is no evidence for those processes that were eliminated by KOIN further suggesting that KOIN is indeed removing false-positive signals.

To extend this analysis to noncoding regulatory regions, we applied Genomic Regions Enrichment of Annotations Tool (GREAT) (20) that correlates ChIP-seq signals additionally in noncoding regulatory regions to corresponding genes followed by GO enrichment analysis (Figure 6b). Biological functions are predicted by GREAT on proximal and distal regulatory regions including a stringent filtering with hypergeometric and binomial statistical models. Consistent with the findings for the SRF TF peaks only in proximity to the TSS (Figure 6a), the KOIN-corrected data revealed an enrichment of GO-terms related to known functions of SRF, for example, actin cytoskeleton dependent processes (23) and leukocyte activation in immune response (12) (Figure 6b). Both processes were identified by these two independent bioinformatics approaches to associate GO-defined biological processes with SRF-binding sites in the genome. At the same time, GO-terms such as ‘mast cell activation’, a process SFR has not been linked to, were eliminated after KOIN-correction. We also evaluated whether peaks called by MACS and SICER would further improve biological interpretation of the data in comparison to analysis including all peaks called only by MACS (Supplementary Figure S6a and b). However, this was not the case. Together these results illustrate that uncorrected ChIP-seq data sets most likely overestimate the enrichment of certain GO-terms and biological processes. The enrichment of GO-terms associated with known functions of SRF in the corrected data set further indicates that the KOIN method sharpens the downstream analysis of ChIP-seq data sets.

## DISCUSSION

Here, we introduce KOIN, a novel approach to increase specificity and signal-to-noise ratio in ChIP-seq data, which at the same time removes false-positive signals derived from ‘hyper-ChIPable regions’ (9). KOIN involves a data normalization step utilizing ChIP-seq data derived from KO samples of the respective TF. KO data sets are the optimal technical control for ChIP-seq experiments, since sheared chromatin is not processed like the target ChIP sample. Furthermore, KO data sets are less prone to overamplification of genomic loci during library construction prior enriched by IgG (3,4,24,25). We tested KOIN with six different ChIP-seq data sets and could recognize a significant reduction of false-positive signals in five out of six data sets. Intriguingly, for one data set (PU.1) with a very low false positive rate, KOIN even improved the recognition of specific peaks due to an improved signal-to-noise ratio. Removing false-positive peaks was also associated with a relative increase of sites with the primary TF-specific binding motif. Reasons for the identification of binding motifs for other TFs instead of the target TF could be caused by variations in TF-binding strength based on modifications of the motif sequence or participating co-factors. For example, weak TF-binding sequences need binding of secondary TFs for DNA binding of the main TF cooperatively resulting in a ChIP-seq peak signal (26). Moreover, as illustrated on the level of metadata analysis, removing false-positive peaks was critical for accurate biological interpretation of genome-wide mapping studies of TFs. In addition to MACS, we also tested the combination of KOIN with SICER, another peak caller for ChIP-seq data. In principle, the combination of SICER with KOIN also removed the large number of false-positive peak signals identified in our analysis. However, irrespective of the used MACS version, MACS performed better overall, particularly considering the downstream metadata analysis. When performing gene ontology enrichment analysis on uncorrected or KOIN-corrected data, a reduction of biological processes was apparent, particularly for those data sets with the largest number of false-positive peaks. As exemplified for the SRF data set, there was no random loss of GO-terms within the GOEA networks established by BINGO but rather a loss of complete subnetworks. This finding further supports the notion that KOIN is not randomly removing signals but rather specifically removes false-positive signals. KOIN-correction also increased the relative fraction of peaks with the primary binding motif for the TF under study. Moreover, these sites showed significantly lower *P-*values despite the reduced number of peaks further underlining specificity of these sites. Based on these findings, we postulate that the binding of TFs might actually be more specific for their primary binding motifs than previously suggested by analysis of WT data sets only. We also hypothesized that KOIN-correction would lead to an elimination of ‘hyper-ChIPable regions’. Indeed, of the 18 ‘hyper-ChIP-able regions’ identified in our WT data sets, we did not find any after KOIN-correction and this was even true for the pioneer TF PU.1. This was somewhat surprising, since it has been recently shown that chromatin accessibility is changed after knockout of pioneer TFs (27). A possible explanation for the removal of ‘hyper-ChIPable regions’ in data sets of knockout pioneer TFs like PU.1 might be compensatory mechanisms of other pioneer TFs, for example, FLI1 enabling the opening of chromatin (28). Hence, we propose that KOIN is a simple and systematic approach to remove such sites in future studies on other TFs. Furthermore, it will also be of great interest to examine whether current standard ChIP-seq approaches targeting novel TFs, chromatin regulators as well as the recently introduced ‘occupied regions of genomes from affinity-purified naturally isolated chromatin’ (ORGANIC) method (29) would also benefit from including KO samples into the analysis. A potential scenario for future studies defining the binding of as yet uncharacterized TFs to DNA might be that newly developed TF-specific antibodies are tested on native chromatin, for example, using ORGANIC profiling in both WT and TF-KO samples, to determine affinity, specificity, signal-to-noise ratio and ‘hyper-ChIPable regions’. Based on this analysis, recommendations for use of KO samples in further studies can then be provided. One limitation of such studies is the requirement for specific KO samples. However, due to the revolutionary developments in genetic engineering (30,31), the development of both mouse and human KO cells for this approach should present a challenge, not being an unsurmountable hurdle. The profoundly improved biological interpretation of the corrected data provides major impetus for such an effort.

## SUPPLEMENTARY DATA

Supplementary Data are available at NAR Online.

SUPPLEMENTARY DATA
