# Supplementary material for: Optimization of transcription factor binding map accuracy utilizing knockout-mouse models
Source: Nucleic Acids Res. 2014 Nov 5;42(21):13051–60. doi: 10.1093/nar/gku1078 (PMC4245947; doi:10.1093/nar/gku1078)
Supplement: SUPPLEMENTARY DATA [file supp_42_21_13051__index.html]

Optimization of transcription factor binding map accuracy utilizing knockout-mouse models — Optimization of transcription factor binding map accuracy utilizing knockout-mouse models — SUPPLEMENTARY DATA 

# Optimization of transcription factor binding map accuracy utilizing knockout-mouse models

## SUPPLEMENTARY DATA

**Files in this Data Supplement:**

- SUPPLEMENTARY DATA
