# Supplementary material for: Optimization of transcription factor binding map accuracy utilizing knockout-mouse models
Source: Nucleic Acids Res. 2014 Nov 5;42(21):13051–60. doi: 10.1093/nar/gku1078 (PMC4245947; doi:10.1093/nar/gku1078)

Supplementary Material

Supplementary Figure 1. Overview of KOIN workflow. Main steps necessary for the generation of false positive curated KOIN peak files are presented with optional steps for downstream data analysis.

Supplementary Figure 2. Mapping quality of ChIP-seq datasets and peak overlap of MACS and SICER called peaks with and without KOIN-correction. **(a)** The number of reads for each uniquely called genomic position in all six datasets is depicted for WT (black bars) and KO (white bars) dataset reads. Corresponding false positive rates determined with KOIN method are visualized in boxes in the top right corner of each bar plot. **(b)** Bar chart of percentages for validated MACS peaks with SICER program for standard and KOIN method.

Supplementary Figure 3. Comparison of peaks called by MACS versus peaks called by MACS and SICER. **(a)** Percentages of tags containing ATF3, GATA3, SRF and PU.1 motifs are depicted for called peaks by MACS (dark colored) or MACS/SICER (light colored) with KOIN (blue bars) or standard method (green bars) for all six datasets. **(b)** Binomial *p*-values for motif enrichments of KOIN called peaks by MACS (dark red) or MACS/SICER (light red) are plotted for all six datasets.

Supplementary Figure 4. Enrichment of TF binding motifs in knockout experiments and motif ratios for wildtype against knockout datasets. **(a)** Enrichment for specific motif PWMs was performed at KO peak sites. Percentages of target sequences with the corresponding top 10 motifs were ranked according to their enrichment *p*-values for six ChIP-seq datasets. Scales for *p*-values and percentages were used according to Figure 4. **(b)** Top 5 enriched motifs in WT (black bars) and KO (white bars) datasets were used to find corresponding motif counts in WT and KO peak files. Motif counts were then normalized to total peak counts and the ratio of WT against KO motif counts was depicted as fold change ratios. Motifs with high abundance in WT datasets show positive values, whereas motifs with higher normalized numbers in KO datasets possessed negative ratio values.

Supplementary Figure 5. Percentage and ranking of TF binding motifs at false positive and knockout sites. Input sequences for motif analysis were only taken from false positive peaks excluded during the KOIN-correction process. Percentages of target sequences with the corresponding motif are depicted for all six datasets and ranked according to their enrichment *p*-values. Top 10 enriched motifs are depicted with corresponding motif sequence. The same scale for *p*-value and percentage was chosen as in Figure 4.

Supplementary Figure 6. MACS peak calling optimizes biological downstream analysis in comparison to SICER. Gene ontology enrichment analysis was performed with GREAT tool for **(a)** $ATF3^{unstim}$ and **(b)** SRF dataset after calling common peaks with KOIN approach MACS/SICER (orange bars) or only with MACS (red bars) program resulting in *p*-values describing the significance of GO-term enrichment.

Supplementary Table 1. Number of peak counts for standard and KOIN method. Peak counts called during standard and KOIN method are displayed corresponding to their genomic locations in the datasets and the relative in- or decrease of peak numbers is listed accordingly.

Supplementary Table 2. ”Hyper-ChIPable regions” found in different datasets after standard method peak calling. Every defined “hyper-ChIPable region” can be found in at least 2 out of 6 used datasets defined by extremely high tag numbers, verified by visual inspection with the UCSC genome browser.

Supplementary Table 3. The top 25 peaks called with KOIN method do not contain ”hyper-ChIPable regions”. Top 25 KOIN-corrected peaks with highest normalized peak counts do not show “hyper-ChIPable” criteria.

```
# KOIN Pipeline (Linux commands)
#
# Created by Wolfgang Krebs, 02. September 2014
# LIMES Institute, University of Bonn, Germany
#
# Calculations were performed on a RedHat 64bit Linux environment.
# Options for corresponding programs might need to be adapted to fit to
# other datasets (program directories, reference genome, genome size, p-
# values...)
#
# General steps:
# 1. Alignment
# 2. Peak Calling (KOIN)
# 3. Optional data analysis
#
# For all described general steps other aligners or peak calling programs
# could be utilized, as long as the peak caller can use a KO dataset as
# background during calculations.
#
# Before usage: Exchange all path descriptions in <> with corresponding
# destinations.
#
# Linux commands are depicted in green
#



### 1. Alignment of ChIP-seq experiments with Bowtie
#
# In this first step, wildtype (WT) and knockout (KO) ChIP-seq experiments
# (e.g. in fastq format) are aligned to the reference genome of choice (e.g.
# mm9/mm10 for mouse datasets)and exported as sam files.
#

<bowtie_directory>/bowtie -t -q -e 70 -l 28 -n 2 --best --maxbts 125 -S
<reference_genome> -q <WT-ChIP-seq-file.fastq> <Aligned-WT-ChIP-seq-
file.sam>

<bowtie_directory>/bowtie -t -q -e 70 -l 28 -n 2 --best --maxbts 125 -S
<reference_genome> -q <KO-ChIP-seq-file.fastq> <Aligned-KO-ChIP-seq-
file.sam>



### 2. Peak Calling using MACS
#
# Knockout implemented normalization (KOIN) is performed during peak calling
# with MACS utilizing the WT dataset as treatment and the KO dataset as
# control resulting in false positive curated peak files in bed format.
#

<MACS_directory>/bin/macs -t <Aligned-WT-ChIP-seq-file.sam> -c <Aligned-KO-
ChIP-seq-file.sam> -n KOIN-corrected-peak-file -f SAM -g 1.87e9 -p 1e-4 -s
51 --bw 150 --on-auto



### 3. Filter out peaks with fold changes <2 for WT/KO tag signals
#
# To further increase specificity of called KOIN peaks, normalized tag
# counts in WT datasets were compared to KO counts for every peak position.
# Peak positions with fold changes <2 for WT/KO tag counts were
```

```
# excluded from downstream analysis. To perform this comparison HOMER
# program was utilized.
#
# First, HOMER required a specific format for ChIP-seq data. Following
# command was used to convert .sam files into HOMER-tag-directories:
#


<HOMER_directory>/bin/makeTagDirectory <WT-dataset-HOMER-tag-directory> -
genome <reference_genome> <Aligned-WT-ChIP-seq-file.sam> -format sam


# Second, normalized ChIP-seq tag counts were counted for every KOIN
# corrected peak site in WT and KO datasets.
#


<HOMER_directory>/bin/annotatePeaks.pl <KOIN-corrected-peak-file.bed>
<reference_genome> -size given -d <WT-dataset-HOMER-tag-directory> <KO-
dataset-HOMER-tag-directory> -noann > <KOIN-corrected-peak-file-WTvsKO.txt>


# Third, peak sites with fold changes <2 for WT/KO tag counts were filtered
# out using SPSS or comparable software.
#



### 4. Optional example steps for downstream data analysis:
#
# a. Annotation of Peak sites with HOMER:
#
# Find nearest transcriptional start sites of known genes for called peaks
#and detailed information about position on the used reference genome.
#

<HOMER_directory>/bin/annotatePeaks.pl <KOIN-corrected-peak-file.bed>
<reference_genome> -size given > <Annotated-KOIN-corrected-peak-file.txt>


#
# b. De novo motif enrichment for KOIN corrected peak sites with HOMER
# program:
#
# Perform a de novo motif enrichment analysis with HOMER at 200bp regions
# around KOIN corrected peaks to detected enriched DNA binding motifs.
#

<HOMER_directory>/bin/findMotifsGenome.pl <KOIN-corrected-peak-file.bed>
<reference_genome> <Motif_calculations_output_directory> -size 200
```

Supplementary Figure 1

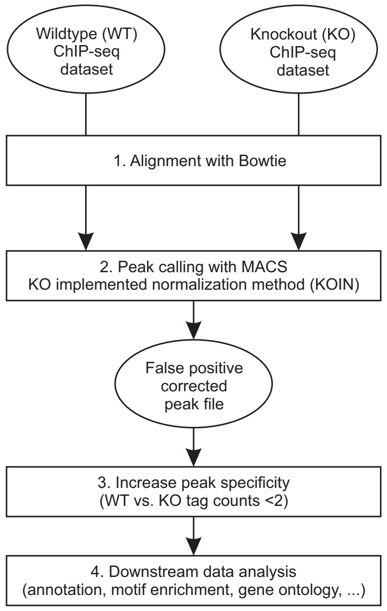

Supplementary Figure 2

a

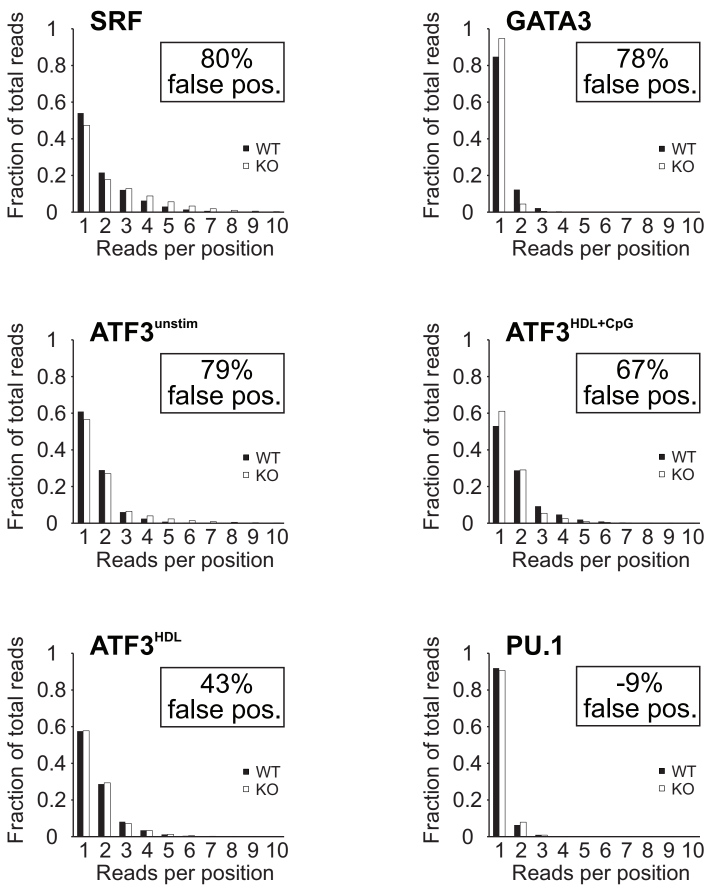

b

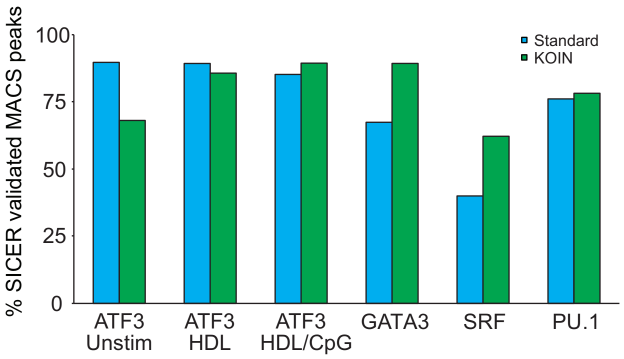

Supplementary Figure 3

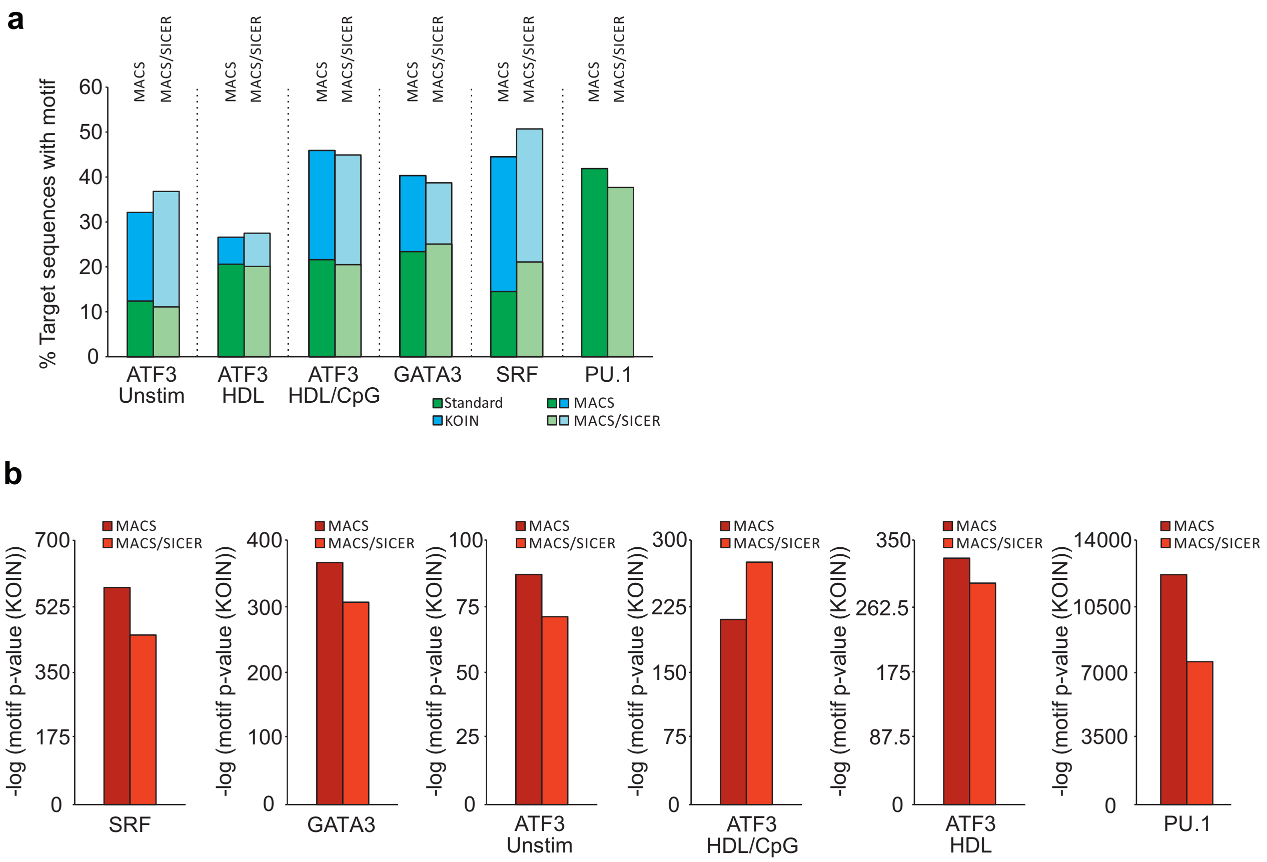

Supplementary Figure 4

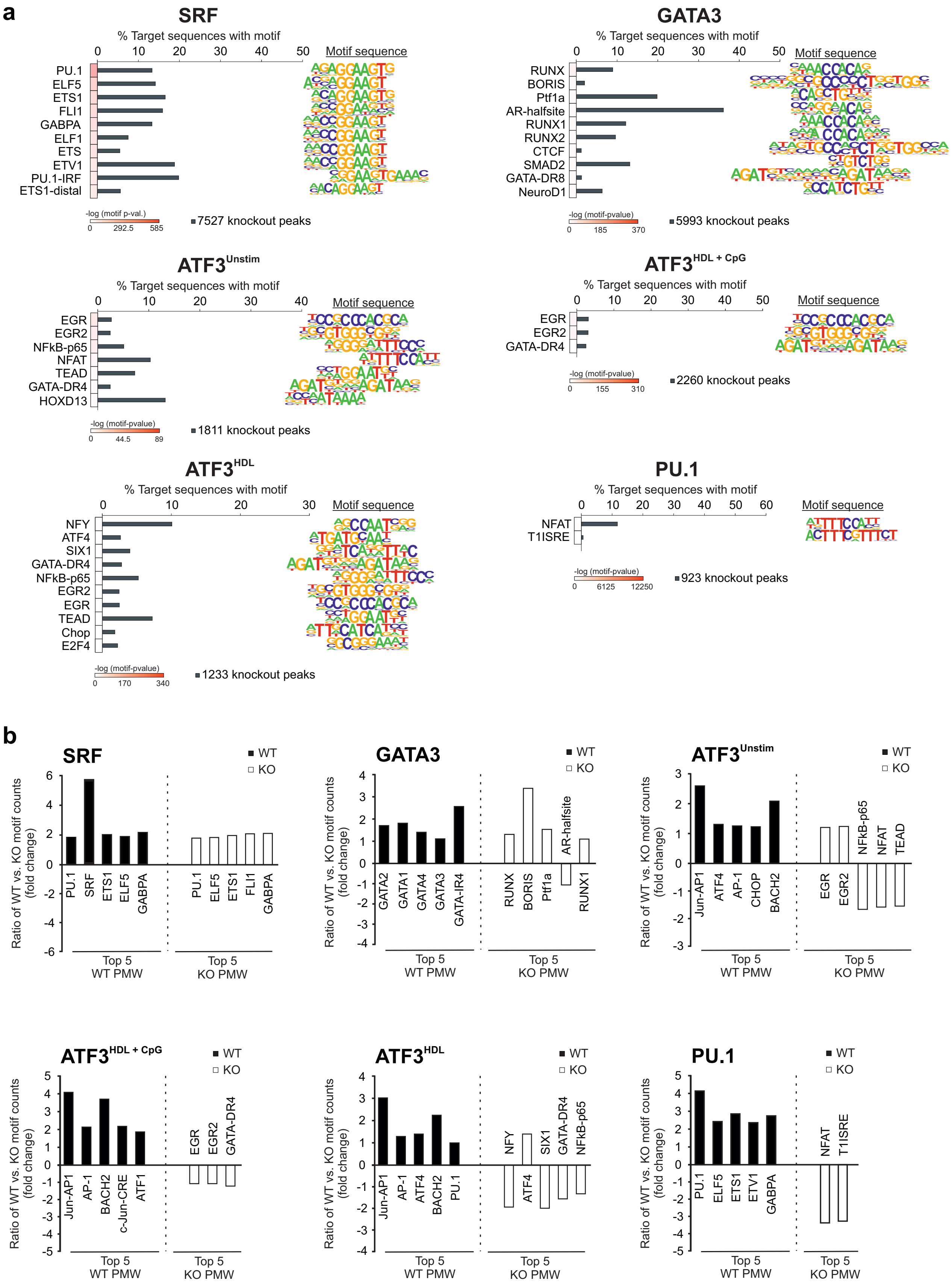

Supplementary Figure 5

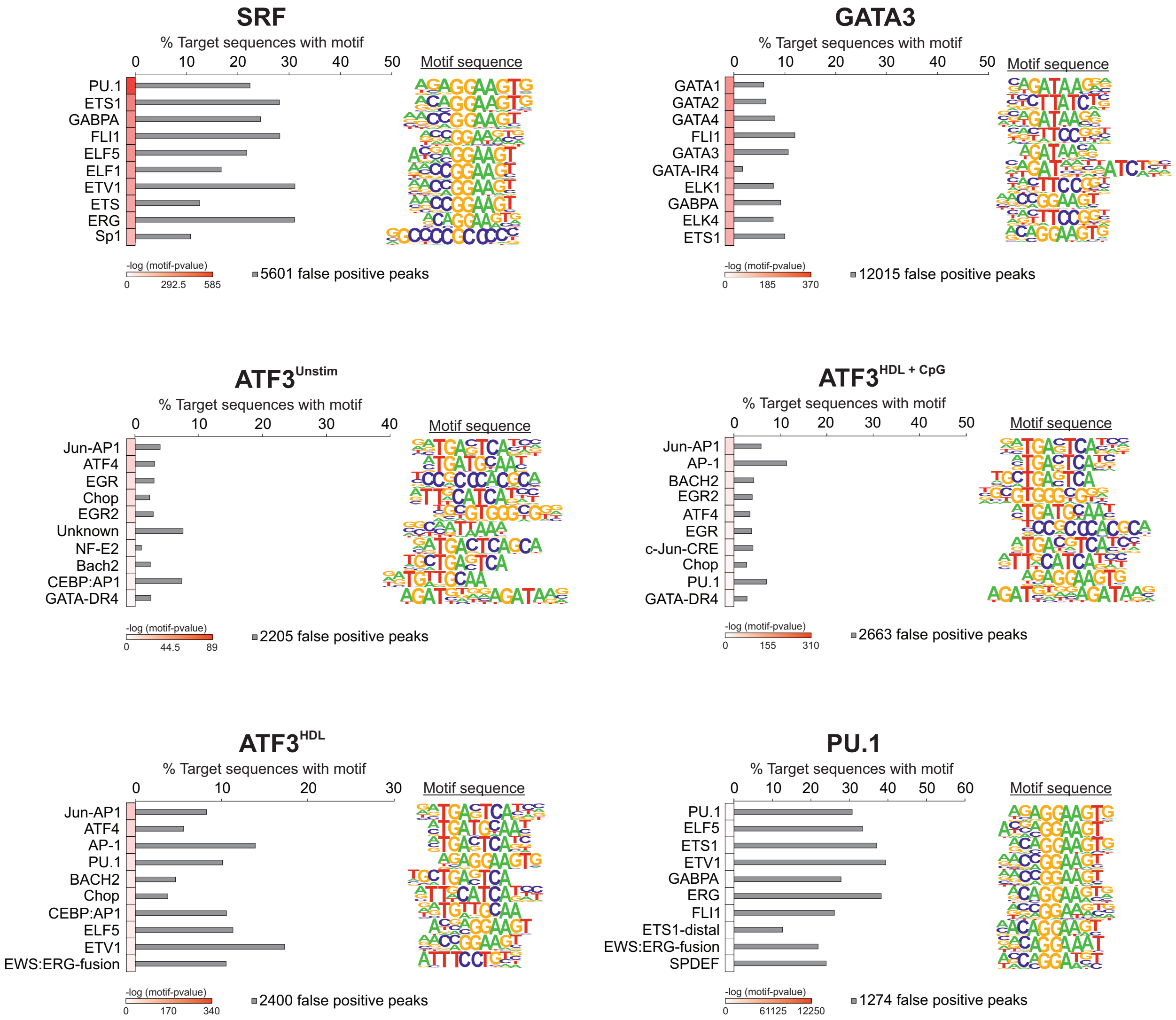

Supplementary Figure 6

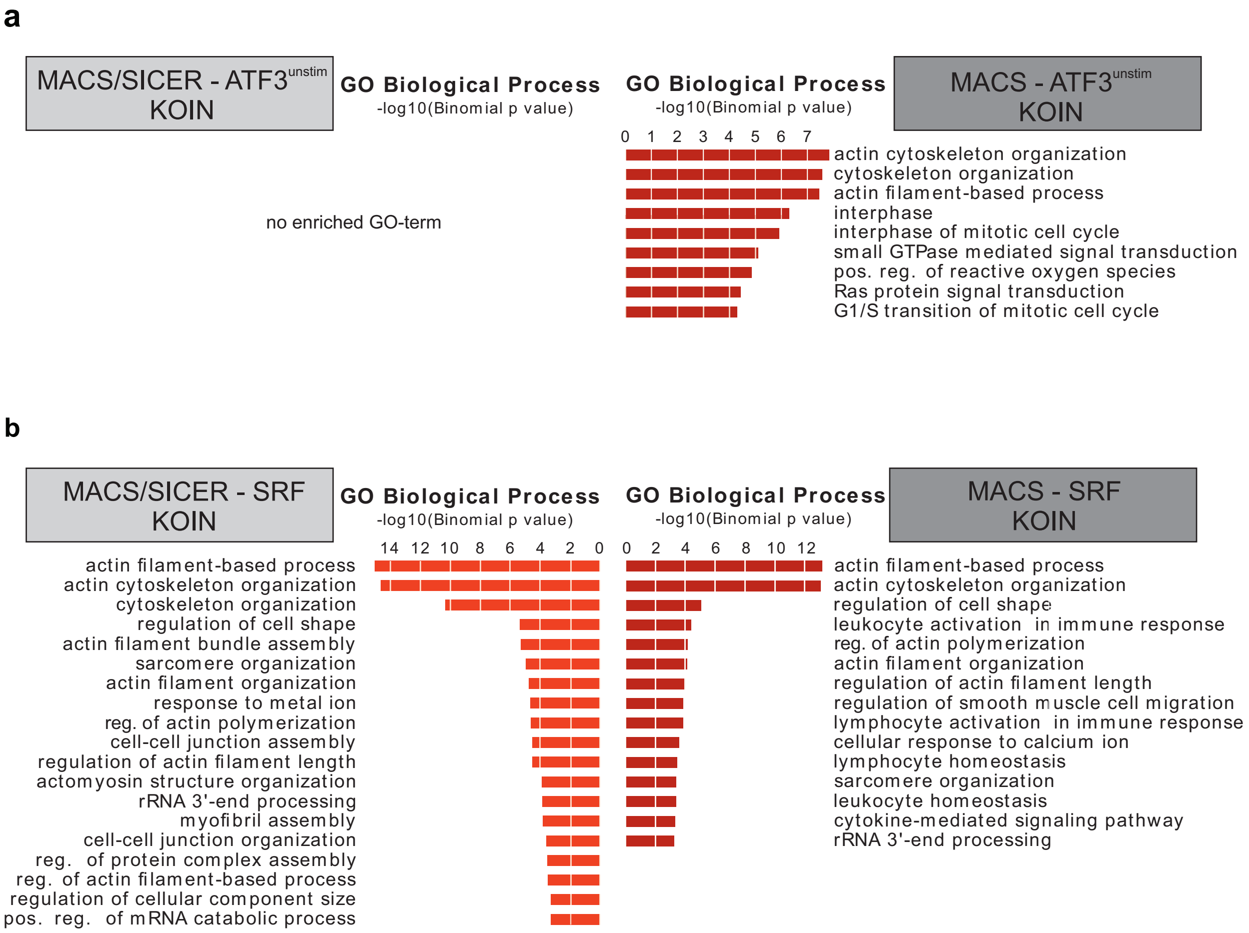

Supplement: SUPPLEMENTARY DATA [file supp_gku1078_nar-02022-met-k-2014-File008.zip › Supplementary Figures.pdf]
